# CYT387, a JAK-Specific Inhibitor Impedes Osteoclast Activity and Oophorectomy-Induced Osteoporosis *via* Modulating RANKL and ROS Signaling Pathways

**DOI:** 10.3389/fphar.2022.829862

**Published:** 2022-03-08

**Authors:** Jing Li, Jiamin Liang, Liwei Wu, Yang Xu, Chengxiang Xiao, Xue Yang, Ran Sun, Jinmin Zhao, Jiake Xu, Qian Liu, Bo Zhou

**Affiliations:** ^1^ Collaborative Innovation Center of Regenerative Medicine and Medical Biological Resources Development and Application, Guangxi Medical University, Nanning, China; ^2^ Guangxi Key Laboratory of Regenerative Medicine, Guangxi Medical University, Nanning, China; ^3^ Research Centre for Regenerative Medicine, Orthopaedic Department, The First Affiliated Hospital of Guangxi Medical University, Nanning, China; ^4^ Southern Theater Air Force Hospital, Guangzhou, China; ^5^ The Second Nanning People’s Hospital, Nanning, China; ^6^ School of Biomedical Sciences, The University of Western Australia, Perth, WA, Australia

**Keywords:** CYT387, osteoclast, ERK, ROS, osteoporosis

## Abstract

Osteoclasts are of hematopoietic lineage and have the ability to degrade mineralized bone tissues. Abnormalities in osteoclastic activity under certain pathological conditions are common in bone diseases such as osteoporosis, osteosclerosis, and arthritis. Although many kinds of drugs are currently used to treat osteoporosis, they have obvious adverse reactions and limitations. CYT387 is a new small-molecule Janus kinase (JAK) inhibitor involved in hematopoiesis, immune modulation, fertility, lactation, and embryonic development. However, it has remained unclear whether CYT387 functionally impacts osteoclast formation. Our study demonstrated through osteoclast formation assay *in vitro*, that the use of CYT387 is a potential drug candidate for treating osteoclast-associated bone disease. The effects of CYT387 on osteoclast formation, bone resorption, NFATc1 activation, and especially intracellular ROS levels were investigated *in vitro*. Further, we examined the preclinical prospects of CYT387 using an oophorectomy (OVX) mouse model of osteoporosis with its anti-osteoclast activity *in vivo*. On the whole, this study shows that CYT387 holds promise for treating osteoclast-related bone illnesses including osteoporosis.

## Introduction

The bone is an extremely dynamic organ that is constantly renewed and reshaped throughout its lifespan, even in adults (Shih and Varghese, 2019). It maintains structural integrity including healthy bone mass and strength, through constant degradation and regeneration (Harmer et al., 2019). Bone remodeling has been recognized to be a dynamic and well-balanced process. When the equilibrium in the osteoblast-mediated bone synthesis and osteoclast-mediated bone resorption is disrupted (Shih et al., 2019), such as in female menopause, decreases in bone mass will occur, accompanied by an increase in structural space within the bone marrow, eventually leading to osteoporosis (He et al., 2012). Osteoporosis is influenced by genetics, endocrine disorders, nutritional deficiency, lifestyle, and other factors (Lou et al., 2018), and poor bone health is much more common among postmenopausal women. With an increase in the senior-citizen segment in the population, the number of osteoporosis patients has been rapidly increasing, placing a heavy economic burden on society.

Osteoprotegerin (OPG) and receptor activator of nuclear factor kappa-B ligand (RANKL) are key effector molecules in osteoclast formation, and the ratio of RANKL and OPG in the microenvironment of the bone is the determining factor for the induction of osteoclast bone resorption (Weitzmann and Ofotokun, 2016). RANK is the cognate receptor of RANKL and a key stimulator of osteoclast (Kearns et al., 2008). OPG can reduce osteoclast differentiation and bone resorption rate by binding to RANKL (Xin et al., 2018), and competing for the binding of RANKL with RANK, thus playing an anti-osteoporosis role. Therefore, the OPG/RANKL/RANK axis is a crucial mechanism regulating osteoclast formation and differentiation (Chao et al., 2019). Two hematopoietic factors, RANKL and macrophage-colony stimulating factor (M-CSF), are known to be key inducers of osteoclastogenesis (Jeong et al., 2018). The recruitment of tumor necrosis factor receptor-associated factor 6 (TRAF6) occurs once RANKL binds to its cell surface receptor RANK. TRAF6 contributes to driving several downstream targets, including NF-κB, NFATc1, and the mitogen-activated protein kinase (MAPK) pathways (Ihn et al., 2015).

Reactive oxygen species (ROS), known as the derivatives of mitochondrial respiratory chains, are known to be involved in apoptosis, inflammatory responses, and cancer (Wan et al., 2020). They stimulate lipid peroxidation and mediates mitochondrial respiratory chain enzyme dysfunction (Campos-Ordonez and Gonzalez-Perez, 2015). Excessive ROS accumulation leads to decreased levels of antioxidant enzymes, and bone destruction. RANKL stimulates the endogenous production of ROS (Yip et al., 2005), which act as important signaling molecules in osteoclast precursor cells (Lee et al., 2005; Yip et al., 2005). NOX1 transfers electrons from NADPH to molecular oxygen, which then catalyzes ROS production (Jianfei et al., 2016). ROS are known to induce many downstream signaling events of osteoclast formation, including MAPK, PI3K, and NF-κB pathways (Condon et al., 2011).

CYT387 is a novel, selective JAK1/2 inhibitor that has been used in clinical trials to treat myelofibrosis (MF) and various cancers of the hematologic system (Abubaker et al., 2014). The Janus Kinase/Signal Transducer and Activator of Transcription (JAK-STAT) pathway performs a critical function in embryogenesis, lactation, fertility, growth, immune regulation, and hematopoiesis (Vainchenker and Constantinescu, 2013). In multiple myeloma (MM), CYT387 prevents interleukin-6 (IL-6)-induced phosphorylation of STAT3 and significantly reduces IL-6 and insulin-like growth factor-1 induced AKT phosphorylation and downstream MEK/ERK pathways, where ERK denotes the extracellular receptor kinase, while MEK denotes mitogen-activated protein kinase (Monaghan et al., 2011). Given the close relationship between bone and bone marrow, there is considerable evidence that blood diseases such as multiple myeloma also have a significant impact on bone health by affecting bone metabolism through a range of cytokines (Gaudio et al., 2020). However, the mechanism of action by CYT387 in osteoclast-associated bone diseases has not been elucidated. Therefore, in this study, we focused on examining the possible preclinical benefits of CYT387 for osteoclast-associated osteolytic illnesses and elucidating the molecular processes that underly the impacts of CYT387 on the formation and function of osteoclast. We concluded that the role of CYT387 in the modulation of osteoclasts may provide guidance for novel therapeutic intervention approaches for patients suffering from osteolytic conditions.

## Materials and Methods

### Reagents

CYT387 (APExBIO Technology, Houston, USA) was dissolved in dimethyl sulfoxide (DMSO) solution at 20 mM concentration for long-term storage and diluted to a working concentration of 20 µM using α-modified minimum essential medium (α-MEM). Penicillin-streptomycin solution, fetal bovine serum (FBS), α-MEM, and DEME were acquired from Gibco (Thermo Fisher Scientific, Waltham, Massachusetts, USA). Recombinant RANKL, as well as recombinant human M-CSF, were purchased from PeproTech EC (London, UK). Cell Counting kit-8 (CCK-8) and E_2_ were acquired from Med Chem Express (Monmouth Junction, New Jersey, USA). The primary antibodies against JNK 1/2 (# 9252S, 1:1,000), P38 (# 8690S, 1:1,000), p-P38 (# 4511S, 1:1,000), p-JNK 1/2 (# 4668S, 1:1,000), p-ERK (# 4370S, 1:1,000) 1/2, P65 (# 4764S, 1:1,000), p-P65 (# 3033S, 1:1,000), IκBα (# 4812S, 1:1,000), HO-1 (# 82206S, 1:1,000), ERK 1/2 (# 4695S, 1:1,000), GSR (# 2622S), SOD2 (# 13141S, 1:100), MMP-9 (# 13667, 1:1,000), and β-actin (# 4970S, 1:1,000) were procured from Cell Signaling Technology (Danvers, MA, USA). Primary antibodies of NFATc1 (sc-7294, 1:100), CTSK (sc-48353, 1:500) and Integrin αV/β3 (sc-7312, 1:100) were procured from Santa Cruz Biotechnology (CA, USA). c-Fos (ab222699, 1:500), ATP6V0D2 (ab236375, 1:1,000), CAT (ab13731, 1:1,000), and GCLC (ab190685, 1:1,000) primary antibodies were procured from Abcam (Cambridge, UK). We also procured all of our secondary antibodies from Cell Signaling Technology, including the mouse and rabbit antibodies (Danvers, MA, USA).

### Osteoclast Culture *In Vitro*

C57BL/6J mice were procured from the Animal Center of Guangxi Medical University (Nanning, China). Femurs and tibias were excised from 6-week-old C57BL/6J mice. To collect bone marrow cells from the femur and tibia, the bone marrow cavity was repeatedly irrigated in a sterile setting using a 1-ml syringe after the epiphyses were resected. Cells were then collected and incubated in a T75 flask in a complete α-MEM medium comprising 10% fetal bovine serum, 25 ng/mL M-CSF, and 1% penicillin-streptomycin solution. After 72 h of culture, the nonadherent cells were removed with phosphate-buffered saline (PBS), and the culture medium was replaced.

Bone marrow-derived macrophages (BMMs) were seeded and incubated overnight in 96-well plates with a density of 6 × 10^3^/well in an incubator, to allow cell adherence. On the second day, after the cells adhered to the bottom, CYT387 was added over a dose gradient of 0, 0.5, 0.75, 1, 1.25 µM. RANKL (50 ng/ml) and M-CSF were utilized to induce osteoclast differentiation as reported (Xu et al., 2000). The medium was replaced every 48 h for 5–7 days until we observed that mature osteoclasts had been formed in the control group. Then, the cells were fixed in 4% paraformaldehyde (PFA) solution for 30 min and rinsed gently twice using PBS, followed by staining using tartrate-resistant acid phosphatase (TRAcP) substrate. Finally, full-hole photographs were taken using Cytation 5 (BioTek Instruments Inc., Winooski, VT, USA), and osteoclasts were defined as cells that had over three nuclei. Quantifications were carried out using ImageJ 1.53 software.

### Cytotoxicity Assay

BMMs were seeded at 8 × 10^3^ cells/well in 96-well plates and subjected to incubation overnight to allow them adhere to the surface of the plates. Following adherence, incubation of the CYT387 to cells was performed for 48 h at different doses (0, 0.5, 0.75, 1, 1.25, 1.5 µM). Afterward, each well received 20 µL CCK-8 solution, followed by incubation for 2 h. Spectrophotometric absorbance or optical density (OD) at a wavelength of 450 nm was measured utilizing a TriStar^2^LB 942 Multimodule microplate meter (Berthold Technologies Gmbh & Co. KG, Baden-Wurttemberg, Germany).

### Podosome Actin Belt Immunofluorescence Staining

To assess the impact of CYT387 on the F-actin-mediated cytoskeletal structure of osteoclasts, BMMs were induced by adding CYT387 (0, 0.5, 1 µM) with 50 ng/ml RANKL until the formation of mature osteoclasts, followed by cells fixing using 4% PFA for 30 min and washing for three times using PBS. Next, the cells were permeated for 5 min utilizing 0.1% Triton X-100 at ambient temperature before being incubated in PBS containing 3% BSA for 30 min continuously. Subsequently, the specimens were transported to a dark place where they were stained with rhodamine-phalloidin and maintained for 1 h. Lastly, the cells were rinsed with appropriate PBS, stained using DAPI (1:100), allowed to settle for 5 min, rinsed thrice again with appropriate PBS, and imaged using Cytation 5.

### Bone Resorption Experiment

The bone slices were numbered on the back with a pencil, soaked in 75% alcohol for 24 h, and then in PBS for 24 h. Finally, they were incubated in α-MEM medium for 48 h before use. BMMs were seeded at a density of 1 × 10^5^ cells/well in 6-well plates and stimulated with RANKL (50 ng/ml) and M-CSF (25 ng/ml). The medium was replaced after every 2 days until small osteoclasts appeared. The digested cells were seeded into 96-well plates at a density of 8 × 10^3^ cells per well, and then treated with 0, 0.5 and 1 µM CYT387, respectively. The control group (without bone slice) and the bone slice group were treated the same way, and the medium was changed every 2 days until the osteoclasts in the control group were fully mature. At this point, the control group cells were fixed using 4% PFA for 30 min and cells were subsequently washed using PBS twice. Then the control group underwent TRAcP staining and osteoclasts counting (TRAcP positive osteoclasts with more than three nuclei). The bone slice group was further cultured for 3–5 days, and then fixed with an electron microscope fixator for 30 min. The surface osteoclasts were gently brushed away with a clean brush, and a scanning electron microscope (SU8100, 3.0 KV) was utilized to record pictures as reported (Wang et al., 2020). The bone resorption area was quantified by ImageJ 1.53 software.

### Osteoblast Culture *In Vitro*

Osteoblast cell line ME3T3-E1 came from Procell Life Science & Technology Co., Ltd. (Wuhan, Hubei, China). MC3T3-E1 osteoblasts were cultured in DEME containing 10% fetal bovine serum and 1% penicillin/streptomycin. Once 90% or more cells adhered to the bottom, the osteogenic basal medium was changed to one comprising 10 mM β-glycerophosphate, 10 nM dexamethasone, and 50 μg/ml ascorbic acid to stimulate osteoblast differentiation. The culture medium was replaced every 3 days thereafter. For osteogenic quantification, the activity of alkaline phosphatase (ALP) was assessed on days 7–10 utilizing an ALP chromogenic kit (Beyotime, Shanghai, China). Images were quantified using ImageJ 1.53 software.

### Quantitative Reverse Transcription-Polymerase Chain Reaction

BMMs were seeded at a density of 1.5 × 10^5^ into 6-well plates, and CYT387 (0, 0.5, 1 µM) was added with RANKL stimulation to induce the BMMs until mature osteoclasts formed. Total RNA was extracted and isolated utilizing TRIzol (Thermo Fisher Scientific), and complementary DNA (cDNA) was obtained with the aid of the reverse transcription kits. MC3T3-E1 cells were seeded at a density of 2 × 10^5^ into 6-well plates, and CYT387 (0, 0.5, 1 μM) was added into the plates. After 7 days, cDNA was extracted and obtained by the same method. The cDNA thus obtained was employed as a template for qRT-PCR in the LightCycler^®^96 system (Roche, Basel, Switzerland). The cycle parameters for PCR were set as follows: 42°C for 60 min, followed by 70°C for 5 min, and then a drop in temperature to 4°C. Target gene expression was computed utilizing the 2^−ΔΔ Ct^ method. CT was acquired through the normalization of the target genes mean value to the cycle threshold (CT) value of β-actin. All primer sequences used for qRT-PCR are listed in Table 1.

**TABLE 1:** The primer sequences of the osteoclast-related genes used in qRT-PCR.

Target gene	Primer sequence (5′–3′)	
	Forward	Reverse
*c-Fos*	CCA​GTC​AAG​AGC​ATC​AGC​AA	AAG​TAG​TGC​AGC​CCG​GAG​TA
*Ctsk*	AGG​CGG​CTC​TAT​ATG​ACC​ACT​G	TCT​TCA​GGG​CTT​TCT​CGT​TC
*Dcstamp*	TCT​GCT​GTA​TCG​GCT​CAT​CTC	ACT​CCT​TGG​GTT​CCT​TGC​TT
*Mmp9*	GAA​GGC​AAA​CCC​TGT​GTG​TGT​T	AGA​GTA​CTG​CTT​GCC​CAG​GA
*Acp5*	TGTGGCCATCTTTATGCT	GTCATTTCTTTGGGGCTT
*Nfatc1*	GGT​GCT​GTC​TGG​CCA​TAA​CT	GAA​ACG​CTG​GTA​CTG​GCT​TC
*Nfe2l2*	GGT​TGC​CCA​CAT​TCC​CAA​AC	TAT​CCA​GGG​CAA​GCG​ACT​CA
*Keap1*	ATGGCGGGGCCCCTAAC	TCA​CGT​CAC​AGA​GTT​GCT​GG
*Homx-1*	GGC​TTT​AAG​CTG​GTG​ATG​GCT	GGC​GTG​CAA​GGG​ATG​ATT​TC
*Cat*	AAG​ATT​GCC​TTC​TCC​GGG​TG	GAC​ATC​AGG​TCT​CTG​CGA​GG
*Gsr*	GCG​TGA​ATG​TTG​GAT​GTG​TAC​C	GTT​GCA​TAG​CCG​TGG​ATA​ATT​TC
*Col1a1*	TGA​AGA​ACT​GGA​CTG​TCC​C	TTT​GGT​GAT​ACG​TAT​TCT​TCC​G
*OPG*	AAT​TGG​CTG​AGT​GTT​CTG​GTG​GAC	TCT​CGT​CTG​GGC​TGA​TCT​TCT​TCC
*Runx2*	GCA​CCC​AGC​CCA​TAA​TAG​A	TTGGAGCAAGGAGAACCC
*β-actin*	TCC​TCC​CTG​GAG​AAG​AGC​TA	ATC​TCC​TTC​TGC​ATC​CTG​TC

### Western Blotting

BMMs were plated into 6-well plates with 5 × 10^5^ cells/well to examine the early impact of CYT387 on osteoclast activation-related signaling pathways. The control and drug groups were pretreated with 1 µM of CYT387 and stimulated for 0, 5, 10, 20, 30, and 60 min with 50 ng/ml RANKL. BMMs were plated into 6-well plates with 1.5 × 10^5^ cells per well, with the attachment of the cells to the plates happening overnight in order to examine the late impacts of CYT387 on osteoclast activation-related signaling pathways. The control group and the drug group (1 µM of CYT387), were stimulated for 0, 1, 3, and 5 days with 50 ng/ml RANKL. Extraction of proteins was performed with RIPA lysis buffer containing phosphatase inhibitors and protease inhibitors; they were then separated by SDS-PAGE and loaded onto a nitrocellulose membrane (Thermo Fisher Scientific, Shanghai, China). For purpose of blocking non-specific immune responses, the membrane was then incubated in 5% skim milk for 1 h at ambient temperature. The primary antibody solution was incubated over the night at 4°C. The next day, after using TBST to rinse the membrane thrice, the secondary antibody was labeled with IRDye fluorescence and the solution was incubated for 1 h at ambient temperature in darkness. The ImageQuant LAS-4000 device (GE Healthcare, Chicago, Illinois, USA) was used to acquire protein bands. Gray value analysis was performed using ImageJ 1.53 software.

### Luciferase Reporter Gene Detection and Immunofluorescence Staining for Nuclear Translocation of NFATc1

To investigate whether CYT387 pre-treatment affects the transcription of NFATc1, the luciferase reporter genes were transfected into RAW 264.7 cells as previously described (Qiu et al., 2021). The transfected cells were subsequently incubated in 48-well plates containing 5 × 10^4^ cells/well and stimulated for 24 h using RANKL (50 ng/ml) and increasing doses of CYT387 (0.5, 1 µM). A luciferase reporter reagent (Promega, Sydney, Australia) was utilized to determine the luciferase activity in accordance with the instructions provided by the manufacturer.

To examine the cellular localization of NFATc1, BMMs were cultured with RANKL (50 ng/ml), and CYT387 (0, 0.5, 1 µM) was added for 48 h, after which the medium was removed. After gently washing with pre-cooled PBS once, they were fixed using 4% paraformaldehyde at ambient temperature for 30 min. Then the cells were permeated for 5 min utilizing 0.1% Triton X-100 at ambient temperature, fixed with 3% BSA for 30 min, and then washed twice with 0.2% BSA solution. Finally, the cells were placed in NFATc1 antibody (diluted in 0.1% BSA solution) and incubated at 4°C overnight. DAPI was used to dye the nuclei, which were then incubated for 5 min, and photographed under a confocal microscope.

### Detection of Intracellular Production of Reactive Oxygen Species

The culturing of BMMs was conducted in the presence of RANKL and M-CSF with varying doses of CYT387 (0.5 and 1 µM) for 48 h. Then 100 µL of dichloro-fluorescein diacetate (DCFH-DA) (10 μM) with α-MEM (without serum) diluted to 1:1,000, was added to each well. At 37°C, 5% CO_2_ was incubated for about 30 min in dark, and ROS was detected utilizing a ROS Assay kit as previously reported (Chen et al., 2019). After that, a fluorescence microscope was utilized to detect dichloro-fluorescein (DCF) at 525 nm emission wavelength and 488 nm excitation wavelength. ImageJ 1.53 software was employed to examine the average fluorescence intensity and the count of ROS-positive cells.

### Determination of Intracellular Calcium Ion Levels

BMMs were plated in 96-well plates at a density of 6 × 10^3^ cells per well in the CYT387 (0, 0.5, 1 µM) medium for 48 h, incubated for 50 min with 4 µM Fluo-4 at a temperature of 37°C, and washed with PBS twice; finally, a fluorescent microscope was employed to determine the intensity of calcium ions at 516 nm emission wavelength and 494 nm excitation wavelength as previously reported (Liu et al., 2019). The intracellular calcium fluorescence intensity was quantified by ImageJ 1.53 software.

### Construction of Oophorectomy-Induced Osteoporosis Model *In Vivo*

The Guangxi Medical University Ethics Committee granted the approval for the construction of an oophorectomy (OVX)-induced osteoporosis model in mice, and all animal experiments were conducted according to the guidelines of the committee. First, 30 C57BL/6J mice (female, aged 11 weeks) were separated into five groups at random (*n* = 6 for each group): sham operation group (Sham), OVX + Vehicle group, OVX + 100 ng/kg of body weight of E_2_ group, OVX + 0.5 mg/kg of CYT387 group (low-concentration group) and OVX + 1 mg/kg of CYT387 group (high-concentration group). Under tribromoethanol anesthesia, mice were operated on either with or without ovariectomy; respectively. One week after surgery, the low-concentration group was given 0.5 mg/kg of CYT387, the high-concentration group was given 1 mg/kg of CYT387, the Vehicle and Sham groups were given an intraperitoneal injection of normal saline, and the E_2_ group was given an intraperitoneal injection of 100 ng/kg of E_2_; the mice were intraperitoneally injected every other day for 6 weeks. Finally, all mice were sacrificed, tibia and femur specimens were harvested for histological evaluation and microscopic computed tomography (micro-CT).

### Micro CT Threshold Analysis

A SCANCO MEDICAL Micro-CT 50 device (SCANCO MEDICAL AG, Switzerland) was utilized to scan the left tibia and identify the region of interest (ROI). An 8 mm slice of the proximal tibia of the ROI was imaged, and three-dimensional reconstruction was performed. CT Analyser1.15.2.2, CTvol 2.3.2.0 and CTvox 3.3.O r1403 were used for data analysis. Bone parameters, such as bone trabecular separation distance (Tb.Sp), bone trabecular thickness (Tb.Th), bone trabecular number (Tb.N) and bone volume fraction (BV/TV) were analyzed and quantified at the selected area of interest 0.5 mm beneath the growth plate.

### Histological Analysis

After sampling, the tibia was fixed in 4% paraformaldehyde for more than 24 h; the tissue was then dehydrated, embedded in paraffin, and finally sliced into continuous sections using a paraffin slicer. Staining of the sections of the kidney, spleen, liver, and heart was carried out utilizing eosin and hematoxylin and the stained sections were scanned utilizing a KF-PRO series automatic digital slice scanner (KONFOONG BIOTECH INTERNATIONAL CO., LTD., Ningbo, China). The images were amplified using K-Viewer software. Tibial sections were stained with HE and TRAcP. Meanwhile, immunohistochemical staining was performed to detect the expressions of CTSK and osteocalcin (OCN). After staining, tibial sections were scanned under a KF-PRO-120 digital section scanner. Finally, in order to examine and analyze the photographs in a consistent manner, QuPath-0.3.0 was utilized.

### Statistical Analysis

Each experiment was performed a minimum of three times, and all experimental results were presented as mean ± standard deviation (SD), or images. The statistical significance of the findings was verified utilizing the student’s *t*-test or the one-way ANOVA test. *p* < 0.05 is defined as the statistical significance unless otherwise stated.

## Results

### CYT387 Attenuates RANKL-Induced Osteoclast Formation *In Vitro*

**FIGURE 1:**
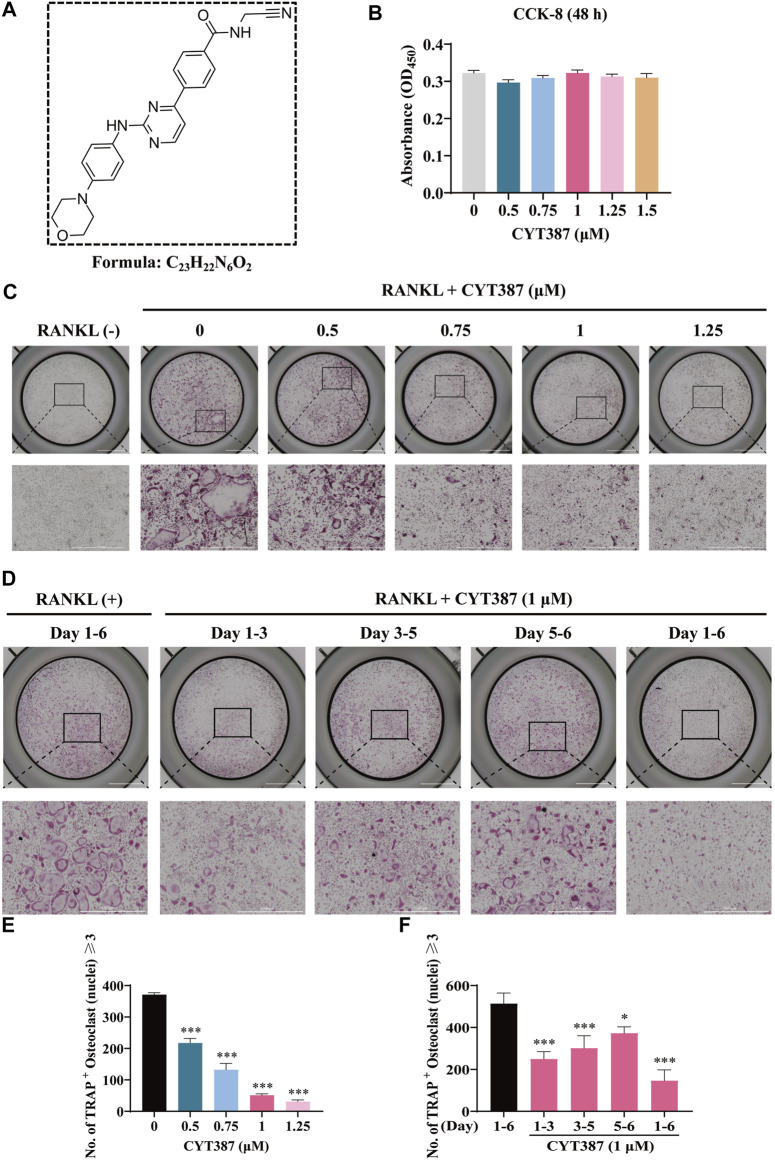
CYT387 suppresses RANKL-induced osteoclastogenesis *in vitro*. **(A)** The chemical structure and molecular formula of CYT387. **(B)** After being treated with different concentrations of CYT387 for 48 h, cell proliferation was detected by a CCK-8 assay (*n* = 3 per group). **(C)** Representative images of TRAcP staining showed that CYT387 inhibited osteoclast formation in a dose-dependent manner after 7 days of 50 ng/ml RANKL stimulation. **(D)** Representative images of TRAcP staining showed time-dependent inhibition of osteoclast formation by CYT387 at 1 µM. **(E)** Quantification of TRAcP-positive cells per well. The data were expressed as mean ± SD (*n* = 3 per group). **(F)** Quantification of TRAcP-positive cells per well when treated with CYT387 in different periods (*n* = 3 per group). The data are shown as the means ± SD. *p < 0.05, **p < 0.01 and ***p < 0.001*.* Scale bar = 2000 μm. BMMs, bone marrow macrophages; CCK-8, cell counting kit-8; RANKL, receptor activator of the nuclear factor-κB ligand; TRAcP, tartrate-resistant acid phosphatase.

The chemical structure of CYT387 is presented in Figure 1A. To clarify whether the cytotoxicity of CYT387 is involved in the inhibition of osteoclast formation, we examined cell viability utilizing CCK-8. As illustrated in Figure 1B, CYT387 showed no effect on BMM cell survival at concentrations of 1.5 µM and below. We further observed whether CYT387 had an effect on osteoclast differentiation. Freshly harvested BMMs were incubated with 50 ng/ml RANKL and CYT387 over a concentration gradient (0.5, 0.75, 1, 1.25 µM). After 7 days, when mature multinucleated osteoclasts were detected in the control group treated with RANKL, the cells were fixed and stained by TRAcP substrate. As displayed in Figures 1C,E, the differentiation of osteoclasts after treatment with CYT387 was significantly inhibited in a concentration-dependent manner, as opposed to the control group. To further determine the stage of osteoclast differentiation at which CYT387 exhibited an inhibitory effect, we treated RANKL-induced osteoclasts with 1 μM CYT387 in a time-dependent manner. We observed a significant reduction in the number of TRAcP-positive cells at 1–3 (early stage) and 3–5 (middle stage) days compared with BMMs stimulated only by RANKL (Figures 1D,F). Together, these observations suggest that CYT387 mainly inhibits early phrase osteoclast differentiation in response to RANKL stimulation.

### Suppression of Osteoclasts Bone Resorption Function *In Vitro* by CYT387

**FIGURE 2:**
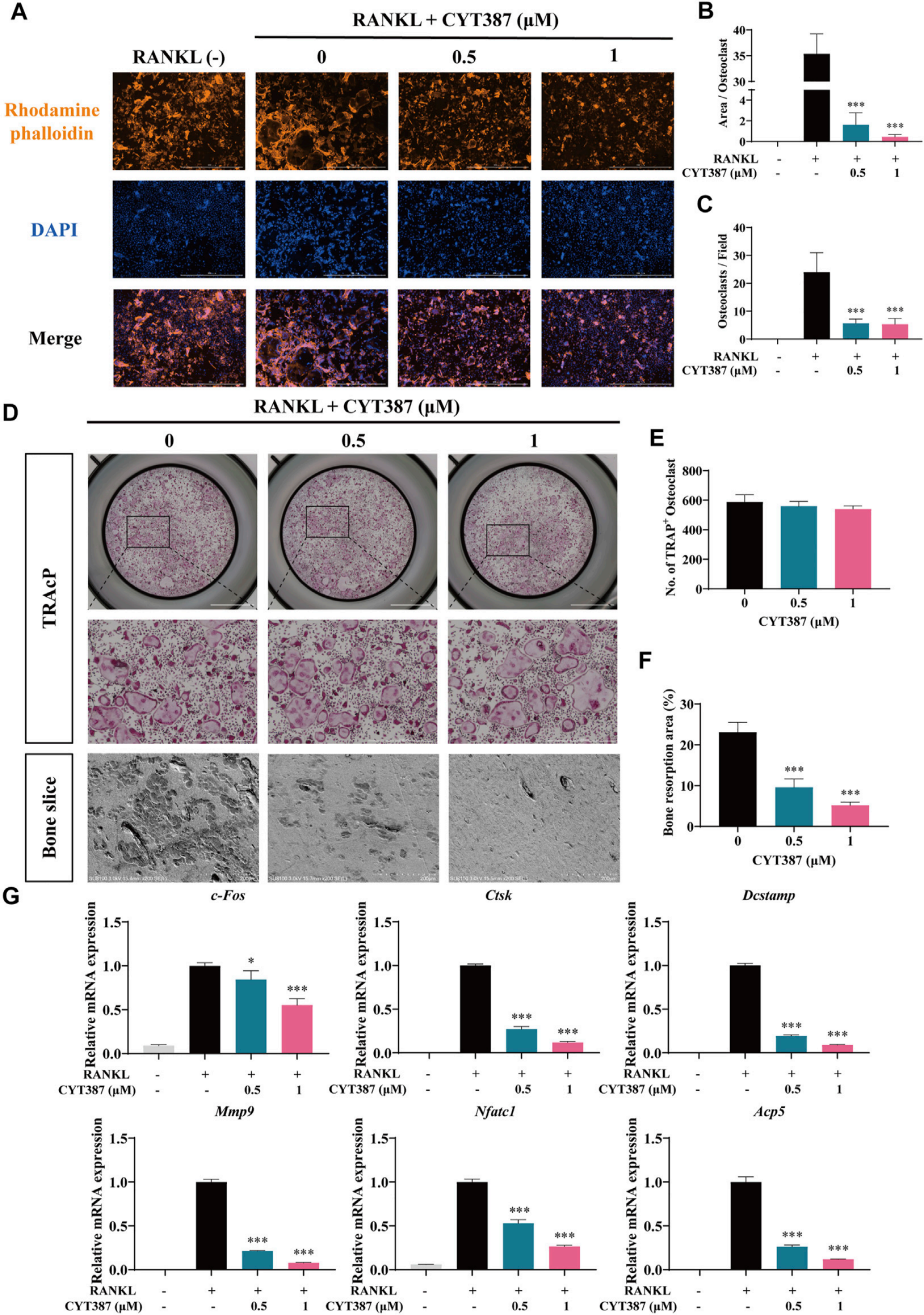
CYT387 impairs the bone resorption function of mature osteoclasts *in vitro* and inhibits the mRNA expression of osteoclast marker genes. **(A)** The representative images of podosome belts in osteoclasts treated with different concentrations of CYT387 were observed by confocal microscopy. Scale bar = 1,000 μm. **(B)** The mean F-actin ring area was quantified (*n* = 3 per group). **(C)** The number of osteoclasts per field was quantified. **(D)** The same number of mature osteoclasts stimulated by 50 ng/ml RANKL were seeded with bone slices, and then the cells were treated with different concentrations of CYT387 to obtain representative bone resorption pit images. The number and area of bone pits were used to evaluate the bone resorption capacity of osteoclasts. Scale bar = 200 μm. **(E,F)** The number of osteoclasts and the total area of bone resorption were evaluated by ImageJ 1.53 software. **(G)** qRT-PCR showed that mRNA expression levels of osteoclast-specific genes, including *c-Fos*, *Ctsk*, *Dcstamp*, *Mmp9*, *Nfatc1* and *Acp5*, were detected in the absence or presence of different concentrations of CYT387. *p < 0.05, **p < 0.01 and ***p < 0.001.

To examine the impact of CYT387 on actin cytoskeleton formation and morphology, we stained osteoclasts with rhodamine-conjugated phalloidin (red), both in the absence and presence of CYT387. After RANKL stimulation, intact podosome belts were formed around mature osteoclasts without CYT387 treatment. In contrast, CYT387 (0.5 and 1 µM) treatment substantially decreased the F-actin area as well as the number of nuclei (Figures 2A–C).

To investigate whether CYT387 affects the bone resorption function of osteoclasts, we seeded an equal number of osteoclasts into bone slices in culture. Quantification of the bone resorption area (%) showed that the proportion of the overall resorption area of osteoclast in the drug group (0.5, 1 µM CYT387) was reduced as opposed to that in the control group (0 µM CYT387) (Figures 2D–F). These results suggest that CYT387 effectively blocks bone resorption in mature osteoclasts.

### Repression of the Levels of Osteoclast-specific Genes Expression by CYT387

Consistently, after CYT387 (0.5, 1 µM) treatment, the expression of osteoclast-related genes, including c*-Fos*, *Ctsk*, *Dcstamp*, *Mmp9, Nfatc1* and *Acp5*, was found down-regulated (Figure 2G). These results provide evidence that CYT387 suppresses osteoclast formation *in vitro* by inhibiting the gene expression of osteoclast-specific markers.

### Suppression of RANKL-Induced NFATc1 Expression and Activation by CYT387

**FIGURE 3:**
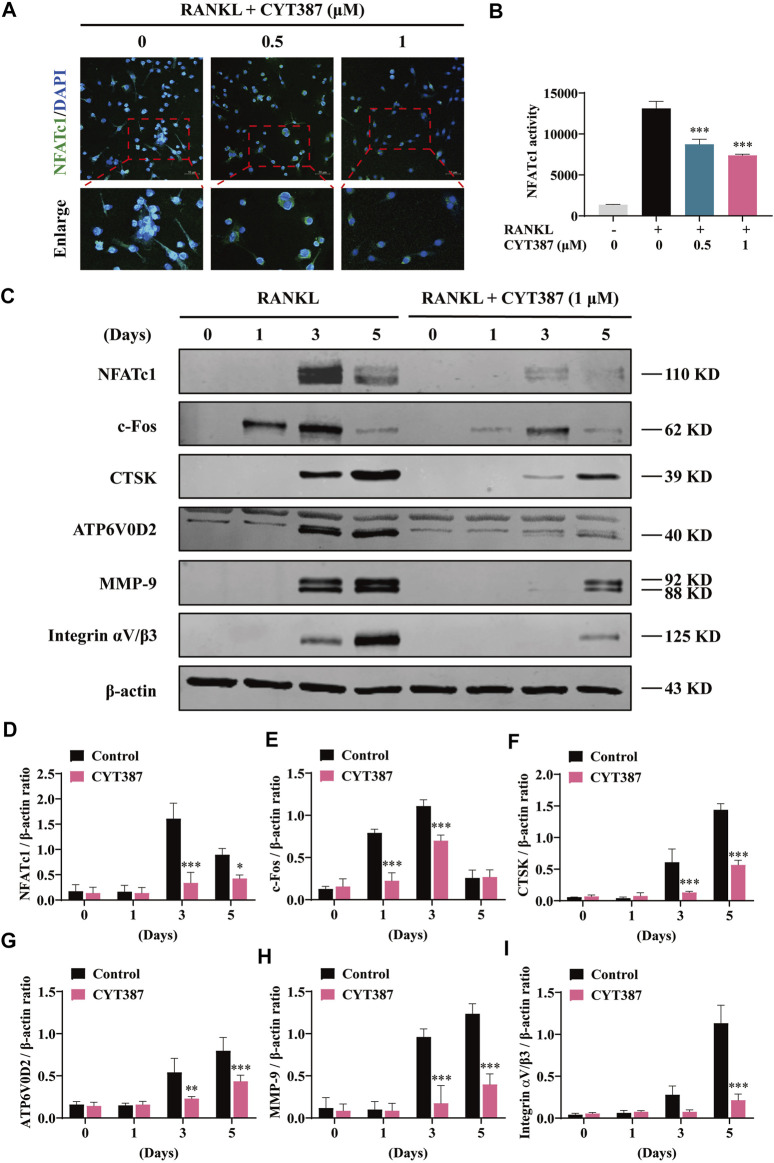
CYT387 inhibits RANKL-induced NFATc1 activation. **(A)** Immunofluorescence images of NFATc1 nuclear translocation following RANKL stimulation without or with (0.5 and 1 μM) CYT387 treatment (Magnification = ×20). Scale bar = 50 μm. **(B)** Transcriptional activity of NFATc1 promoter was detected by the luciferase reporter gene. **(C)** After treatment with CYT387 (1 μM) for 0, 1, 3, and 5 days after RANKL (50 ng/ml) stimulation, western blot was used to detect the expression of RANKL downstream signaling protein. **(D–I)** Quantification of the ratios of band intensity of NFATc1, c-Fos, CTSK, ATP6V0D2, MMP-9 and Integrin αV/β3 relative to β-actin. All experimental data are expressed as mean ± SD. *p < 0.05, **p < 0.01 and ***p < 0.001*.*

In addition, treatment with CYT387 significantly reduced the nuclear translocation of NFATc1 (Figure 3A). Next, we measured the transcriptional activity of NFATc1 using RAW264.7 cells with the luciferase reporter gene. The results showed that the activity of NFATc1 was decreased obviously after CYT387 (0.5, 1 µM) pretreatment compared to the control (Figure 3B). In accordance with the results of the qRT-PCR analysis shown in Figure 2G, after RANKL stimulation, CYT387 significantly inhibited the expression of NFATc1 and c-Fos, as well as CTSK, ATP6V0D2, MMP-9 and Integrin αV/β3 by western blot analyses (Figures 3C–I).

### Inhibition of RANKL-Activated MAPK Signal and Intracellular Ca^2+^ Influx by CYT387

**FIGURE 4:**
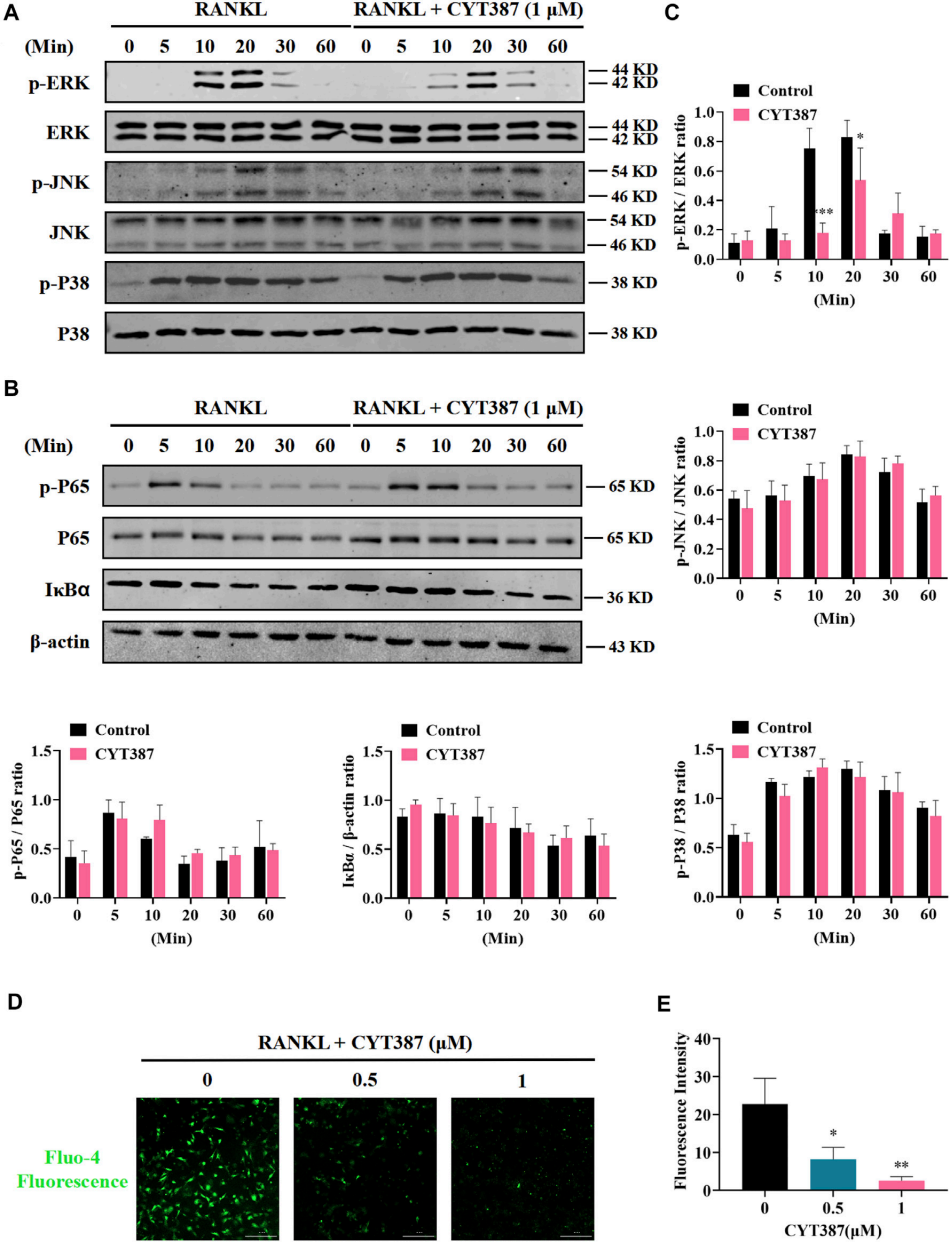
CYT387 negatively regulates osteoclast formation and bone resorption by inhibiting MAPK signaling. **(A)** In the early stage of osteoclast formation, CYT387 inhibited ERK degradation but did not inhibit the phosphorylation of JNK and P38 MAPKs. Total cell protein was extracted with BMM pretreated with 1 μM CYT387 for 1 h, and then stimulated with 50 ng/ml RANKL for a specific time. Protein expression and phosphorylation status of ERK, JNK, and P38 were assessed using specific antibodies. **(B)** CYT387 did not significantly inhibit IκBα degradation and P65 phosphorylation. Protein expression and phosphorylation status of P65 and IκBα were detected using specific antibodies. **(C)** The ratio of phosphorylated p-ERK, p-JNK, p-P38, p-P65 to the corresponding total protein band strength and the ratio of IκBα to β-actin were quantitatively determined. **(D)** Representative images of intracellular Ca^2+^ were obtained 48 h after RANKL (50 ng/ml) stimulation with or without CYT387 (0.5, 1 μM) using the Fluo-4 assay kit. **(E)** The fluorescence intensity of intracellular Ca^2+^ was measured. Scale bar = 300 μm. All experimental data are expressed as mean ± SD. *p < 0.05, **p < 0.01 and ***p < 0.001*.*

It is well known that downstream of the RANK signaling pathway, the MAPK signaling pathway composing of three major subfamilies, P38 kinase (P38), C-Jun N-terminal kinase (JNK1/2), and ERK1/2, displays an integral function in regulating osteoclast development. Therefore, we further investigated whether these signaling pathways play a role in CYT387 suppression of the formation of osteoclast induced by RANKL. Western blot illustrated that the ratio of phosphorylated ERK to total ERK decreased significantly after treatment with CYT387 for 10 and 20 min, respectively. While CYT387 treatment inhibited active phosphorylation of ERK, it had no significant effect on the JNK or P38 signaling pathways (Figure 4A). In addition, no significant inhibition of IκBα degradation or P65 phosphorylation by CYT387 was observed (Figure 4B). These findings suggest that CYT387 inhibited MAPK signaling, especially ERK activity, but did not alter NF-κB signaling. Furthermore, we observed that the intracellular Ca^2+^ fluorescence intensity during the formation of osteoclasts was significantly reduced after the intervention of CYT387 (0.5, 1 µM) (Figures 4D,E).

### Reduction of RANKL-Induced Intracellular ROS Levels and Increase in Expression of Antioxidant Enzymes in BMMs by CYT387

**FIGURE 5:**
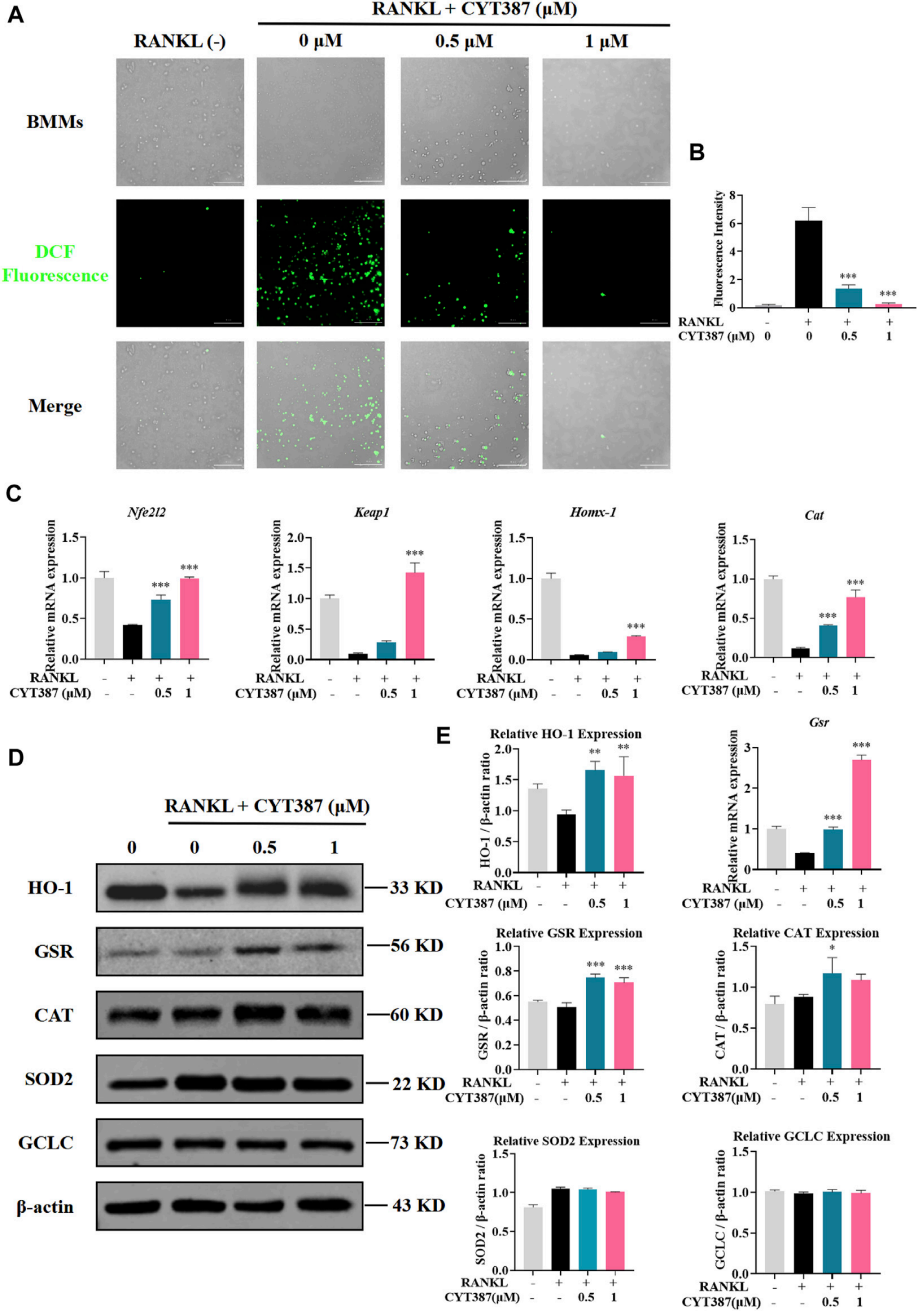
CYT387 reduces RANKL-induced ROS production during osteoclastogenesis. **(A)** RANKL-induced osteoclast formation was treated with different concentrations of CYT387 (0, 0.5, 1 μM), and the representative images produced by ROS were detected by DCFHDA. Scale bar = 300 µm. **(B)** Quantification of the number of ROS-positive cells per field (*n* = 3 per group). **(C)** qRT-PCR was used to detect the expression of antioxidant enzymes *Nfe2l2, Keap1, Homx-1, Cat* and *Gsr* in BMMs treated by RANKL and CYT387 for 48 h. **(D)** Representative Western Blot images of the effects of CYT387 on the expression of antioxidant enzymes, including HO-1, GSR, CAT, SOD_2_ and GCLC. **(E)** Quantification of the ratios of band intensity of HO-1, GSR, CAT, SOD_2_ and GCLC relative to β-actin (*n* = 3 per group). All experimental data are presented as mean ± SD. *p < 0.01, **p < 0.01 and ***p < 0.001*.*

To investigate whether ROS levels are down-regulated by CYT387 during osteoclast differentiation, we used oxidation-sensitive fluorescent dye H2DCFDA to observe ROS levels. The fluorescence signal of DCF, the ROS oxidation product of this dye, was detected and imaged by BioTek confocal microscopy. BMMs were stimulated with RANKL and M-CSF simultaneously, and it was observed that the fluorescence intensity of DCF was reduced in a dosage-dependent way after CYT387 treatment (0.5, 1 µM) (Figures 5A,B). Therefore, our results suggest that CYT387 inhibited osteoclast formation by diminishing ROS activity.

Under normal physiological conditions, ROS clearance mainly depends on a variety of antioxidant enzymes; therefore, we scrutinized the expressions of key antioxidant enzymes, such as heme oxygenase-1 (HO-1), glutathione disulfide reductase (GSR), and catalase (CAT). As shown in the figure, the expression of Nrf2, Keap1, HO-1, CAT and GSR decreased after RANKL stimulation compared with the control group, while the expression of these genes showed an upward trend after CYT387 treatment (Figure 5C). In addition, western blot analysis showed that the protein levels of HO-1, GSR and CAT decreased by RANKL stimulation were restored after CYT387 treatment (Figures 5D,E). Taken together, CYT387 may effectively inhibit RANKL-induced osteoclast differentiation by diminishing ROS production and enhancing the expression of ROS scavenging enzymes.

### Prevention of Bone Loss in Oophorectomy Mouse Models by CYT387

**FIGURE 6:**
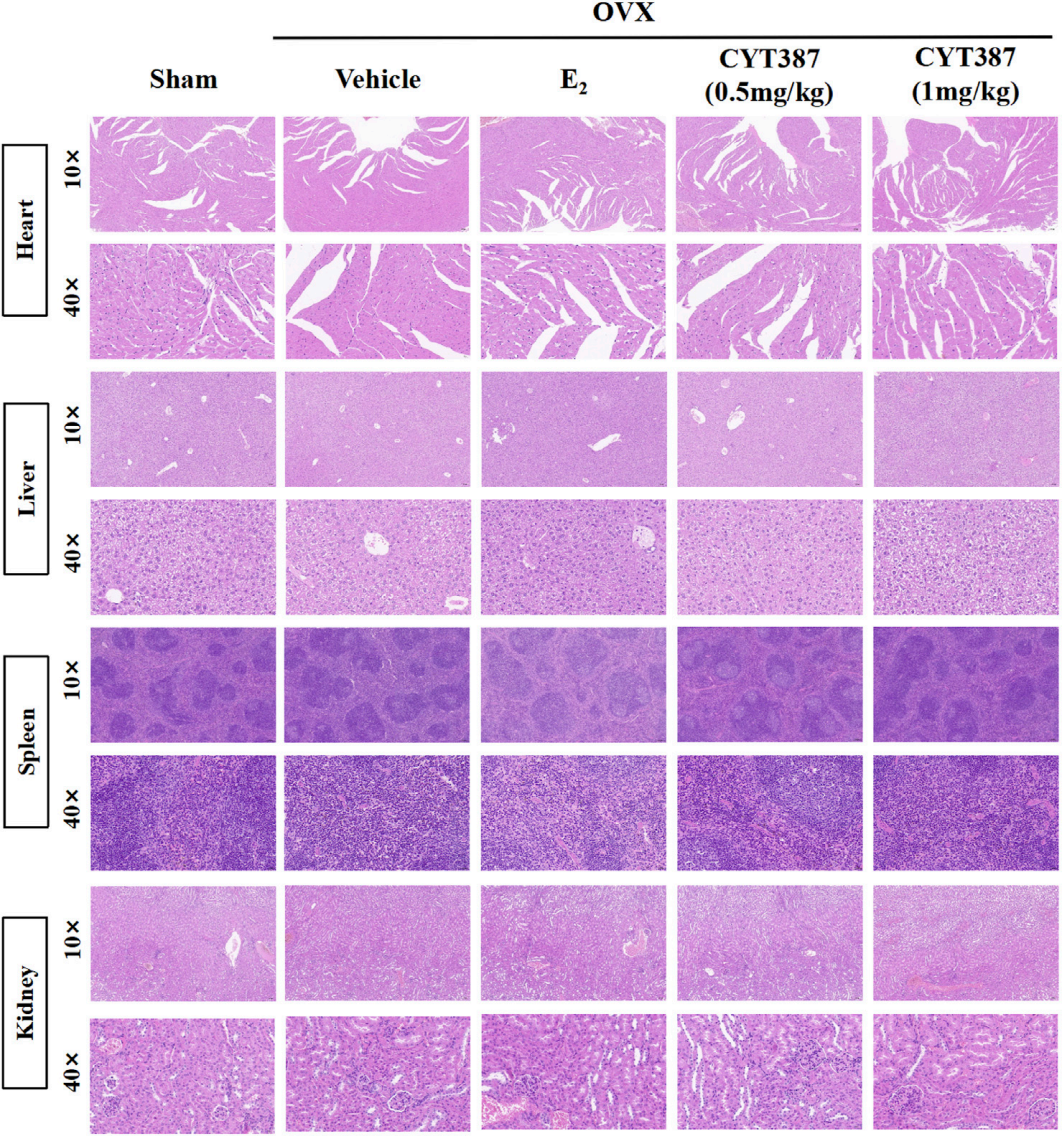
CYT387 has no significant toxic effects on heart, liver, spleen and kidney. Representative images of hematoxylin-eosin staining of the heart, liver, spleen and kidney. 10× multiple scale bar = 100 μm. 40× multiple scale bar = 20 μm.

**FIGURE 7:**
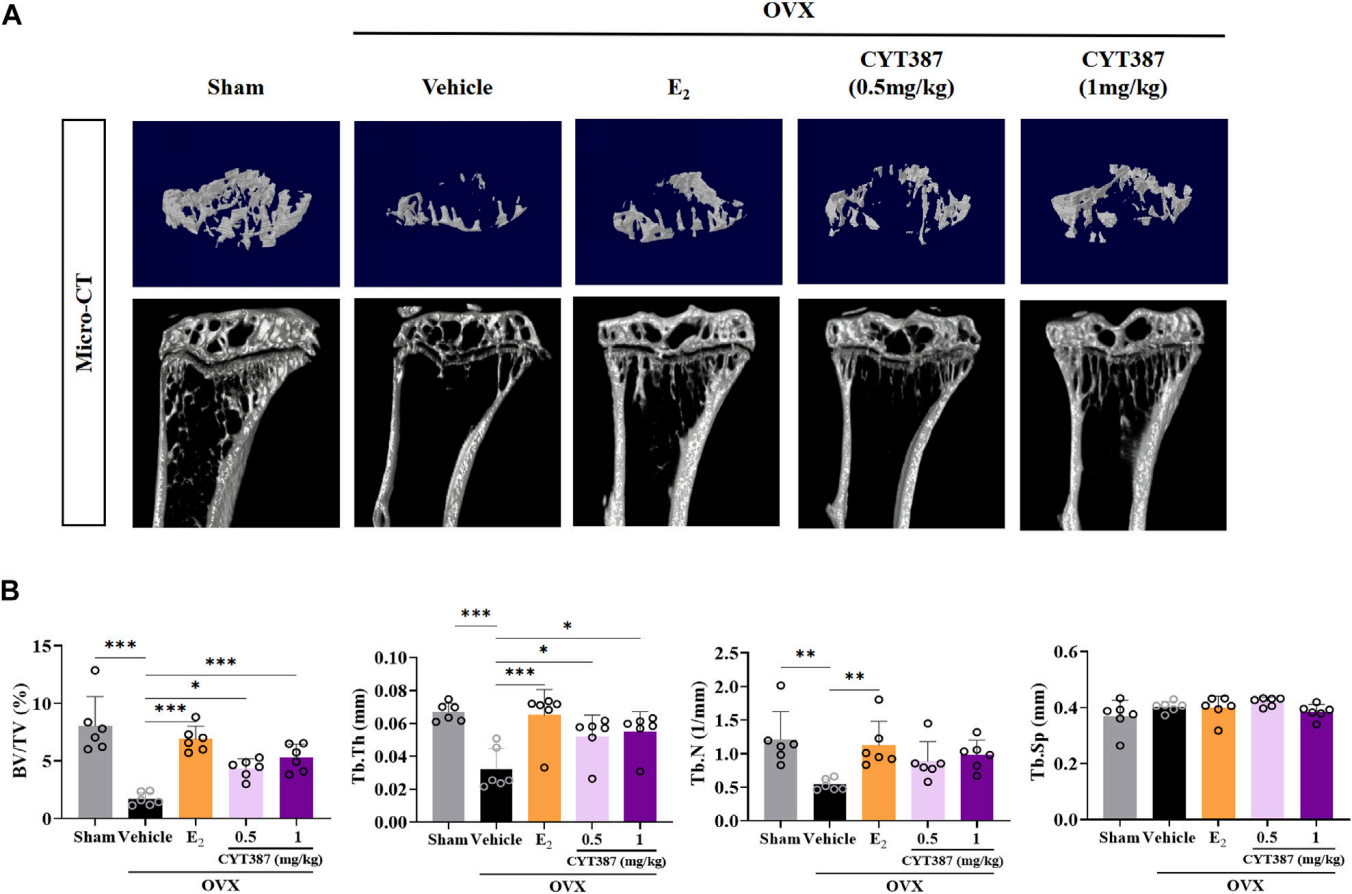
CYT387 treatment can partially reverse bone destruction in ovariectomized mice. **(A)** 3D reconstruction of representative Micro CT images of the proximal tibia in each group. **(B)** The following bone structure parameters were evaluated by quantitative analysis: bone volume to total volume ratio (BV/TV), trabecular thickness (Tb.Th), trabecular number (TB.N), and trabecular separation (Tb.Sp) (*n* = 6 per group). All experimental data are expressed as mean ± SD. *p < 0.05, **p < 0.01 and ***p < 0.001*.*

**FIGURE 8:**
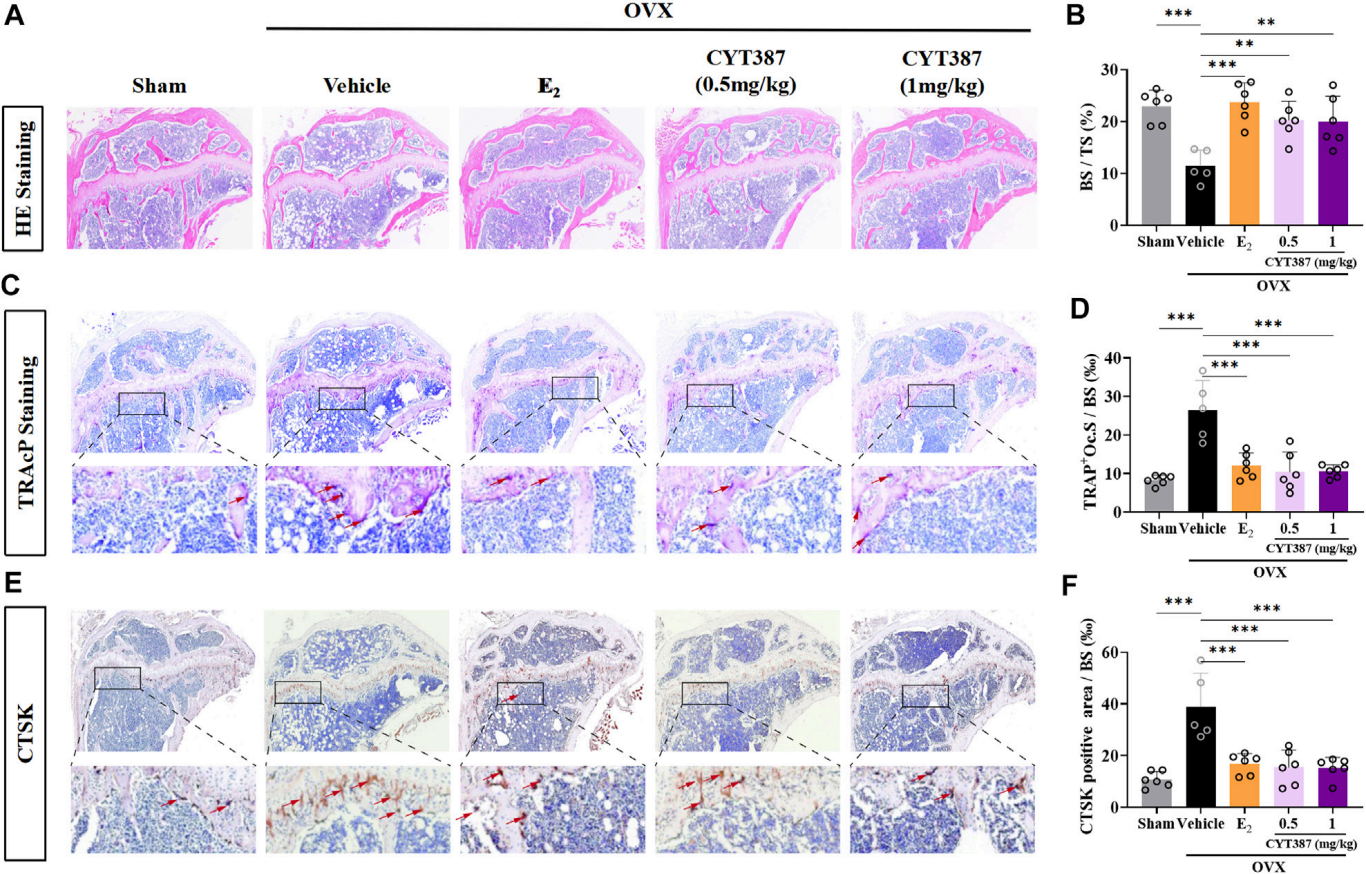
CYT387 ameliorates osteoclast activity in a mouse model of osteoporosis induced by ovariectomy. **(A)** Representative images of histological analysis of femur stained with HE. **(B)** Bone trabecular percentage (BS/TS) was quantitatively analyzed (*n* = 6 per group). **(C)** Representative images of histological analysis of femur stained with TRAcP. The red arrows indicate TRAcP-positive osteoclasts. **(D)** TRAcP-positive cell area per trabecular surface (TRAcP^+^ Oc.S/BS) was quantitatively analyzed (*n* = 6 per group). **(E)** CTSK-positive cells were marked as brownish yellow. **(F)** The level of CTSK was quantitatively measured in the tibia (*n* = 6 per group). All experimental data are expressed as mean ± SD. *p < 0.05, **p < 0.01 and ***p < 0.001*.* OVX, ovariectomized.

To further explore the potential therapeutic effect of CYT387, we simulated a systemic osteoporosis mouse model with OVX. At 6 weeks after OVX or Sham surgery, mice were intraperitoneally injected with E_2_ (100 ng/kg), CYT387 (0.5 or 1 mg/kg) or saline after every 2 days. No major adverse events (including death, infection, and weight loss) were reported during OVX surgery and CYT387 drug therapy. As CYT387 is a small-molecule anticancer drug, we tested by HE staining whether its long-term administration would have an impact on mouse viscera; the results illustrated that CYT387 exhibited no significant toxic effects on kidney, spleen, liver, and heart (Figure 6). Micro CT findings illustrated that CYT387 and E_2_ exhibited a strong protective effect against OVX-induced bone loss (Figure 7A). Micro CT quantitative analysis of the proximal tibia confirmed that treatment with CYT387 reduced the degree of trabecular bone loss compared to untreated OVX (Figure 7B). Examination by HE staining further confirmed the improvement in bone volume after CYT387 treatment. Bone trabecular volume was substantially elevated in the CYT387 group as opposed to the OVX group. In addition, this improvement after CYT387 treatment is accompanied with a decrease in the number of TRAcP-positive osteoclasts in long bones (Figures 8A–D). Similarly, immunohistochemical staining showed that treatment with CYT387 also reduced the area of CTSK positive cells (Figures 8E,F). As expected, the administration of CYT387 protected estrogen deficiency-induced bone loss *in vivo*.

### No Effect of CYT387 in Osteoblast Differentiation

**FIGURE 9:**
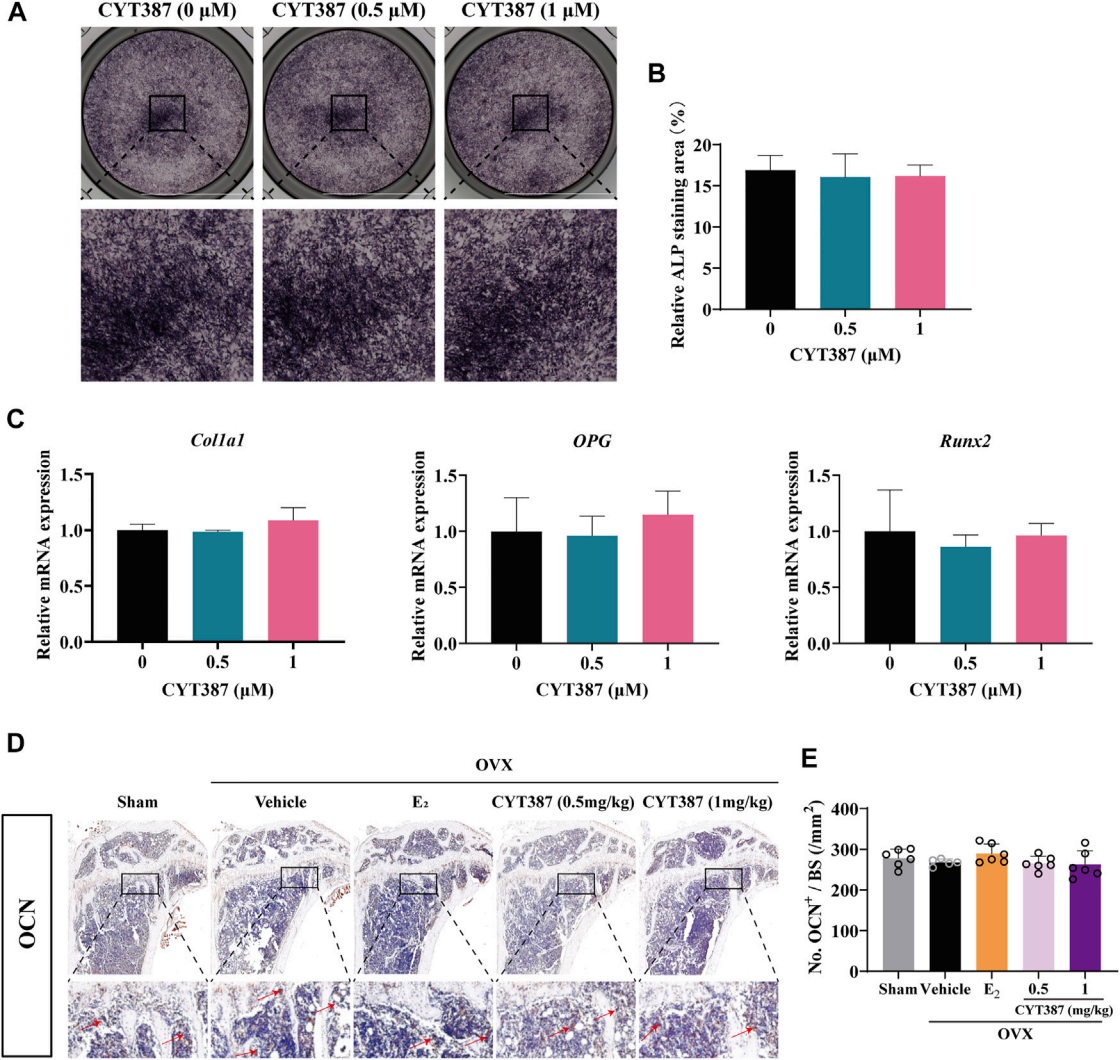
CYT387 does not affect osteoblast differentiation *in vitro* or *in vivo.* **(A,B)** Representative images of Alkaline Phosphatase staining and quantification of BMSCs (*n* = 3 per group). **(C)** qRT-PCR showed that mRNA expression levels of osteoblast-specific genes, including *Col1a1*, *OPG* and *Runx2*, were detected in the absence or presence of different concentrations of CYT387. **(D,E)** The expression and quantification of OCN in proximal tibia were detected by immunohistochemical staining (*n* = 6 per group). All experimental data are expressed as mean ± SD. *p < 0.05, **p < 0.01 and ***p < 0.001*.*

Considering that osteoblasts also play another important role in bone remodeling, we evaluated the impact of CYT387 on the formation of osteoblast utilizing ALP staining. Our results suggested that CYT387 did not affect osteoblast differentiation *in vitro* (Figures 9A,B). QRT-PCR results showed that the mRNA expressions of *Col1a1*, *OPG* and *Runx2* did not change significantly after CYT387 treatment compared with the control group (Figure 9C). Immunohistochemical staining of osteocalcin (OCN), a specific marker of osteoblasts, was performed *in vivo*. Our results showed CYT387 treatment did not affect the expression of OCN in long bones of mice, in line with the findings of *in vitro* experiments (Figures 9D,E).

## Discussion

Osteoporosis is a chronic systemic metabolic disorder associated with bone metabolism imbalance, bone mass decline, and bone microstructure degeneration (Li et al., 2019). It is a disease with one of the highest incidences worldwide among the elderly population. Under normal physiological conditions, the maintenance of bone homeostasis is achieved by osteoclast-mediated bone resorption and osteoblast-mediated bone formation (Ho et al., 2015). Osteoclasts have the ability to degrade bone tissue (Yu et al., 2019), and the formation and activation of abnormal osteoclasts represent a major cause in osteoporosis. Conventional drugs currently used to treat osteoporosis have poor clinical efficacy or obvious gastrointestinal side effects. Currently, more and more small molecule inhibitors have been used for the treatment of various bone-related diseases (Qin et al., 2012; Wang et al., 2017). Several JAK inhibitors such as Tofacitinib, Baracitinib and Phagotinib have been developed for the treatment of rheumatoid arthritis (RA), but there are few reports about JAK inhibitors for the treatment of osteoporosis (O’Gorman et al., 2017). In this research, we illustrated for the first time that CYT387 influences the formation of osteoclast and bone resorption *in vitro* by inhibiting the RANKL-induced ERK signaling pathway, and by inhibiting ROS levels. Our studies also show that CYT387 blocks the development of OVX-induced osteoporosis *in vivo*.

Activation of the receptor RANK by RANKL is an important initiating signal for osteoclast formation (Zarb et al., 2019), via the adaptor protein TRAF6. TRAF6 stimulates and activates the downstream NF-κB and MAPK pathways by recruiting and activating TAK1 (Verron et al., 2010). The classic NF-κB signaling pathway encompasses the stimulation of the IKK complex that phosphorylates and degrades IκBα *via* ubiquitin-dependent proteasome (Kong et al., 2015), resulting in NF-κB translocation to the nucleus to initiate transcriptional activity. The MAPK family, such as P38, JNK1/2, and ERK1/2 (Bouchemal et al., 2017), is closely associated with RANKL-induced osteoclast differentiation. The binding of RANKL to its receptor RANK results in the recruitment of the connector protein TRAF6, which then triggers ERK activation. ERKs phosphorylate many downstream transcription factors such as c-Fos to control osteoclast formation (Lee et al., 2018). Our study found that treatment with CYT387 significantly inhibited ERK phosphorylation, with no significant effects on IκBα degradation or P65 phosphorylation was observed.

As a key transcription factor in osteoclast formation (Murata et al., 2017), NFATc1 in osteoclasts is automatically amplified to maintain stable expression, and its induction and transcriptional activity are induced by RANKL. This results in the nuclear translocation of NFATc1 and synergistic up-regulation of related osteoclast specific marker genes, including *Ctsk*, *Dcstamp*, *Mmp9*, and *Acp5*, with osteosarcoma oncogene (*c-Fos*) and other transcription factors (Min et al., 2018), eventually forming mature osteoclasts. NFATc1 is in turn regulated by intracellular calcium oscillations *via* calcineurin (Huang et al., 2010). Notably, NFATc1-deficient osteoclast progenitor cells could not differentiate into osteoclasts (Hyun-Ju Kim et al., 2014), and NFATc1-deficient mice developed a severe osteoclast phenotype (Jeong-Hoon Kim et al., 2014). It is widely established that the proto-oncogene c-Fos is induced by epidermal growth factor (EGF) stimulation, and is a critical element of the transcription factor complex that activates the protein AP-1 (Peng et al., 2016). Consistently, our findings illustrated that CYT387 reduced the transcriptional activity of NFATc1 by inhibiting the activity of c-Fos and intracellular calcium activity, resulting in reduced nuclear translocation of NFATc1.

During osteoclastic bone resorption, MMP-9 is required for early migration of osteoclasts and resorption by mature osteoclasts, which can promote the formation of osteoclast ruffled border (Ganguly et al., 2020). Vacuolar ATPases (V-ATPases) regulate the secretion of H^+^, protease dissolution of crystalline hydroxyapatite and the organic matrix (Uluçkan et al., 2009). CTSK is a key protease in osteoclast bone resorption, mainly responsible for the degradation of collagen and other matrix proteins (Xue et al., 2015). CTSK-deficient mice showed enhanced bone tissue mineralization and improved bone remodeling (Kegelman et al., 2020). Our results showed that CYT387 inhibited MMP-9, ATP6V0D2, and CTSK expression, in line with its inhibition in osteoclast function.

ROS, as a new signaling medium involved in apoptosis, cell growth, differentiation, and inflammatory response, is considered the second messenger that determines the fate of cells and acts on a variety of signaling molecules (Bigarella et al., 2014). Cellular ROS mainly include free radicals, such as singlet oxygen hydrogen peroxide (H_2_O_2_), hydroxyl radicals, and other non-free radical oxides (Parvez et al., 2018). O^2−^ is the first product of the mitochondrial respiratory chain that is then swiftly transformed to H_2_O_2_ and decomposed by superoxide dismutase (SOD), which can then be reduced to water by glutathione peroxidase or catalase (CAT). Studies have shown that NADPH oxidase (NOX1) is the main producer of ROS after RANKL stimulation (Lee et al., 2010), and other cytokines or external stimuli such as hypoxia, TNF-α, EGF, and IL-6 also stimulate ROS production. Under normal physiological conditions, ROS levels in cells are relatively stable. Overconsumption or accumulation of ROS affects many signaling pathways, leading to cellular dysfunction (Zhang et al., 2016). Osteoclasts adapt to oxidative stress by upregulating a series of antioxidant enzymes, including CAT, glutathione-disulfide reductase (GSR), SOD2, Heme oxygenase-1 (HO-1), and the γ-glutamylcysteine synthase catalytic subunit (GCLC). HO-1, a downstream enzyme of the nuclear transcription factor Nrf2, was found to play a key role in inhibiting the response of osteoclast precursors to RANKL (Florczyk-Soluch et al., 2018). CAT, as an important ROS detoxifying enzyme, participates in the decomposition of intracellular hydrogen peroxide and maintains normal ROS levels (Glorieux and Calderon, 2017). GSR also plays an important role in scavenging ROS and protecting bones from ROS (Bakirezer et al., 2019). Current literature indicates that the activation of MAPK is mainly mediated by H_2_O_2_ (Lin et al., 2009). Lee et al. provided evidence that oxidant scavenger action on ROS is parallel to MAPK activation and osteoclast differentiation. Elevated ROS levels may be responsible for MAP kinase activation, which in turn stimulates osteoclast formation (Lee et al., 2005). Data from this study suggest that CYT387 inhibits the differentiation of osteoclast by up-regulating the expression of the antioxidant enzymes, thus inhibiting intracellular ROS.

Based on these *in vitro* results, we established an OVX mouse model to investigate whether CYT387 has potential *in vivo* therapeutic effect on osteoporosis. Our results demonstrated that the trabecular structure was significantly improved. Moreover, the degree of trabecular bone loss, as well as the area of TRAcP-positive osteoclasts, were substantially lowered in the CYT387 treated group. CTSK staining showed a significant increase in the total area of CTSK-positive cells in bone trabecular bone of OVX mice, whereas treatment with E2 or CYT387 showed a significant decrease. Thus, it may be concluded that CYT387 protected mice from OVX-induced bone loss.

**FIGURE 10:**
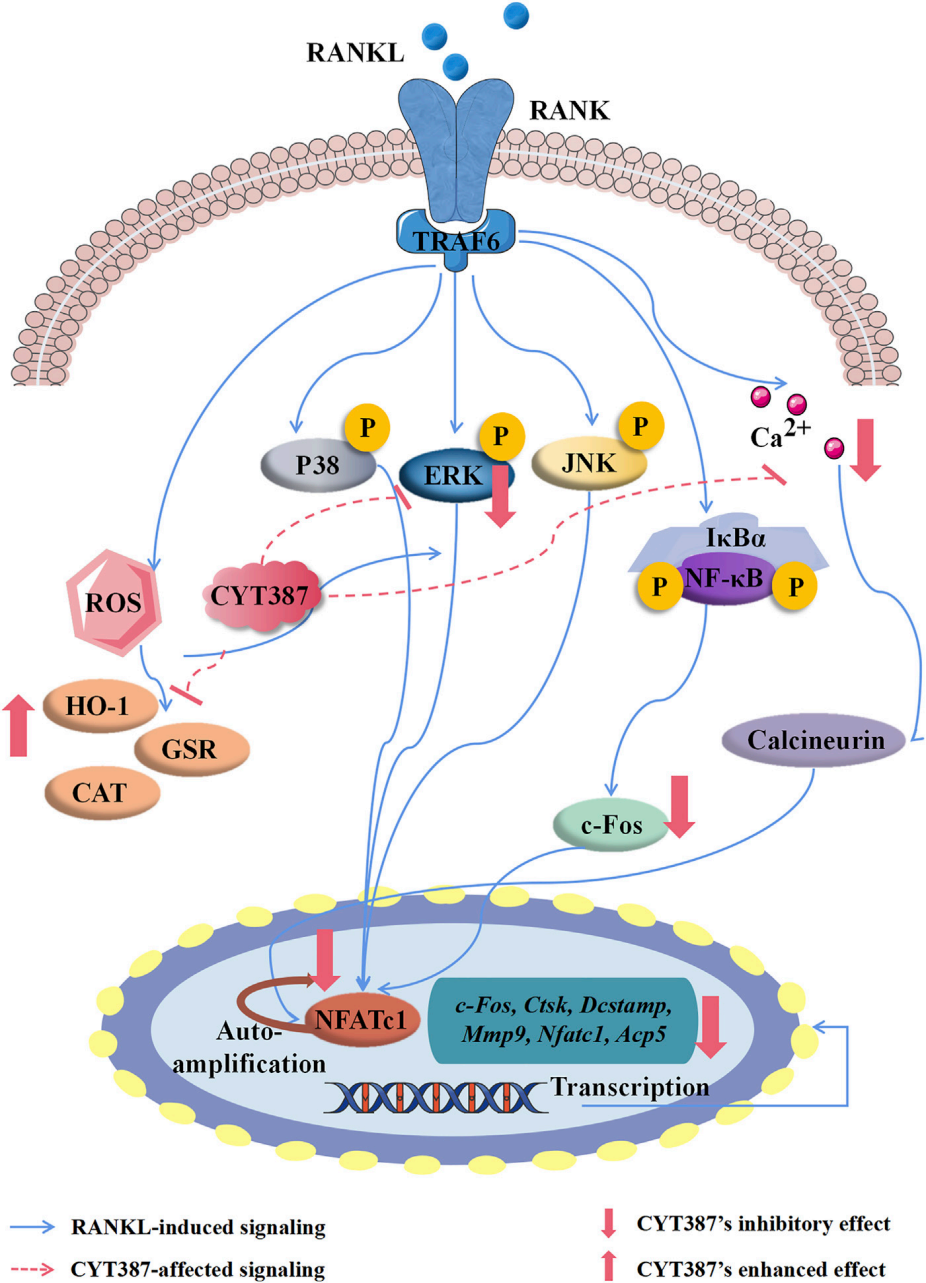
A proposed scheme for the inhibition of CYT387 in osteoclastogenesis. Our results demonstrate for the first time that CYT387 inhibits the level of ROS and RANKL-induced signaling pathways, accompanied by increased expression of antioxidant enzymes, thereby inhibiting the formation and function of osteoclasts. NFATc1, nuclear factor of activated T cells 1; *c-Fos*, Proto-oncogene C-Fos; *Ctsk*, cathepsin K; *Dcstamp*, dendritic cell-specific transmembrane protein; *Mmp9*, matrix metallopeptidase 9; *Acp5*, tartrate resistant acid phosphatase; RANKL, receptor activator of nuclear factor-κB (NF-κB) ligand; NF-κB, nuclear factor-κB; MAPKs, mitogen-activated protein kinases; ROS, reactive oxygen species.

In conclusion, this study discovered that CYT387 inhibited ROS levels *in vivo* by enhancing the expression of the antioxidant enzymes and impeded other RANKL signaling pathways, leading to impaired osteoclast differentiation and function *in vitro* and *in vivo* (Figure 10). Further, we observed that CYT387 had no effect on osteoblast differentiation. On the whole, the study suggests that CYT387 may provide a promising treatment option for bone diseases caused by excessive osteoclastic bone resorption.

## Data Availability

The original contributions presented in the study are included in the article/Supplementary Materials, further inquiries can be directed to the corresponding authors.
